# The Role of Glucocorticoids in Inflammatory Diseases

**DOI:** 10.3390/cells10112921

**Published:** 2021-10-28

**Authors:** Sybille D. Reichardt, Agathe Amouret, Chiara Muzzi, Sabine Vettorazzi, Jan P. Tuckermann, Fred Lühder, Holger M. Reichardt

**Affiliations:** 1 Institute for Cellular and Molecular Immunology, University Medical Center Göttingen, 37073 Göttingen, Germany; sybille.reichardt@med.uni-goettingen.de (S.D.R.); agathe.amouret@med.uni-goettingen.de (A.A.); chiara.muzzi@med.uni-goettingen.de (C.M.); 2 Institute of Comparative Molecular Endocrinology, Ulm University, 89081 Ulm, Germany; sabine.vettorazzi@uni-ulm.de (S.V.); jan.tuckermann@uni-ulm.de (J.P.T.); 3 Institute for Neuroimmunology and Multiple Sclerosis Research, University Medical Center Göttingen, 37075 Göttingen, Germany; fred.luehder@med.uni-goettingen.de

**Keywords:** glucocorticoid receptor, multiple sclerosis, inflammatory bowel disease, asthma, acute lung injury, rheumatoid arthritis, graft-versus-host-disease, nanoparticles

## Abstract

For more than 70 years, glucocorticoids (GCs) have been a powerful and affordable treatment option for inflammatory diseases. However, their benefits do not come without a cost, since GCs also cause side effects. Therefore, strong efforts are being made to improve their therapeutic index. In this review, we illustrate the mechanisms and target cells of GCs in the pathogenesis and treatment of some of the most frequent inflammatory disorders affecting the central nervous system, the gastrointestinal tract, the lung, and the joints, as well as graft-versus-host disease, which often develops after hematopoietic stem cell transplantation. In addition, an overview is provided of novel approaches aimed at improving GC therapy based on chemical modifications or GC delivery using nanoformulations. GCs remain a topic of highly active scientific research despite being one of the oldest class of drugs in medical use.

## 1. Introduction

Inflammatory diseases constitute one of the major health problems in modern society. They are caused by a pathologically aberrant immune system that either attacks its own cells (autoimmunity) or reacts to harmless foreign antigens (allergy). In other cases, such as acute lung injury (ALI) and COVID-19, the immune response is inappropriately strong. Regardless of the antigen, pathological inflammation results in tissue damage, which can restrict the quality of life and may culminate in lethal multiorgan failure. Autoimmune diseases such as multiple sclerosis (MS), inflammatory bowel disease (IBD), and rheumatoid arthritis (RA), as well as allergic diseases such as asthma and contact dermatitis are highly prevalent, especially in western countries [1]. Although there is a plethora of therapies which can be used to slow down the progression of inflammatory diseases, including various monoclonal antibodies [2], no other treatment is as successful as glucocorticoids (GCs). As a matter of fact, GCs are the most widely prescribed class of drugs worldwide and their efficacy in the treatment of acute or chronic inflammation is undisputed [3,4]. The development of synthetic GCs with improved potency and specificity has so improved the management of inflammatory diseases that these days it is difficult to imagine being without them. However, GCs are not perfect: treatment with them may be accompanied by a plethora of side effects, including osteoporosis, muscle wasting, and hypertension [5]. In addition, GCs induce hyperglycemia and promote the development of type 2 diabetes by increasing hepatic gluconeogenesis and downregulating IRS-1 in fat and muscle which causes insulin resistance [6]. Hence, there is still an urgent need to improve this therapeutic regimen.

Inflammatory responses involve a large variety of immune cells such as T and B cells, macrophages, dendritic cells (DCs), granulocytes, and NK cells, which are responsible for antigen recognition and pathogen clearance. However, cells of non-hematopoietic origin also contribute to the inflammatory cascade in target organs, for instance epithelial cells in the intestinal tract or the airways and fibroblast-like synviocytes in joints [7,8,9]. Thus, it is a demanding task to find out which of these cell types are critical for the pathogenesis of each individual disease. Often, mice with specific gene disruptions have been used to identify relevant cell types in preclinical models [10], information which then forms the basis for the development of tailored therapies involving chemically modified GC derivatives [11] or novel GC nanoformulations [12]. Although animal models do not perfectly reflect the clinical situation encountered in humans, this caveat is often outweighed by the power of genetics, which makes it possible to delete or modify genes by homologous recombination in embryonic stem cells or using CRISPR/Cas9 technology. Ultimately, a better understanding of how GCs interfere with inflammation in individual diseases will help to optimize available therapies, thereby leading to more efficient treatment regimens with fewer side effects.

## 2. Molecular Mechanisms of GC Action

The majority of GC activities are mediated by the ubiquitously expressed GC receptor (GR, NR3C1), which is found in the cytosol in an inactive form associated with heat shock proteins [13]. The mineralocorticoid receptor (MR, NR3C2) also binds GCs but it is only expressed in certain tissues [14]. Upon entering cells, GCs become locally activated by 11β-hydroxysteroid dehydrogenase 1 (11β-HSD1), an enzyme that converts cortisone to cortisol or deoxycorticosterone to corticosterone [15]. In contrast, GCs are inactivated in cell types expressing 11β-hydroxysteroid dehydrogenase 2 (11β-HSD2), for which reason aldosterone then becomes the main ligand of the MR despite its lower serum concentration. Since macrophages co-express the GR and MR but lack 11β-HSD2, GCs engage both receptors in these cells, contingent upon their concentration [16].

**Figure 1:**
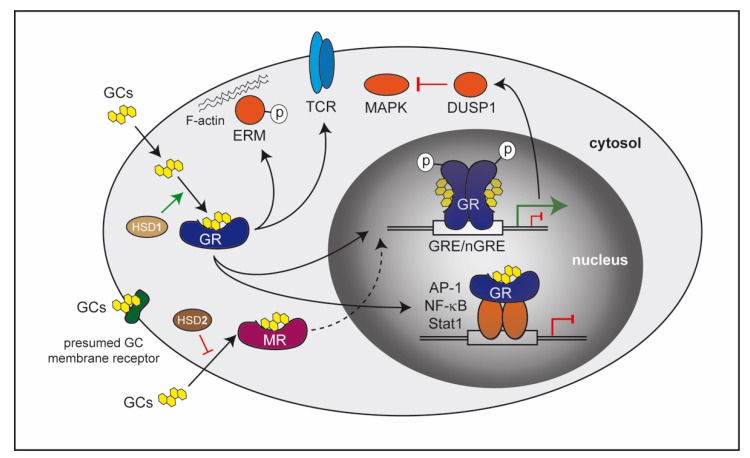
Molecular mechanisms of GCs involved in the control of inflammation. Natural and synthetic GCs can passively pass the cell membrane due to their lipophilic nature and bind to the GR localized in the cytosol. Here, GCs can be activated by the enzyme 11β-HSD1, but they may also be inactivated in some cell types by 11β-HSD2. The ligand-bound GR can directly exert its activity in the cytosol by inducing phosphorylation of ERM proteins interacting with F-actin or influence T-cell receptor (TCR) signaling. In most cases, however, the GR translocates into the nucleus, where it binds to GREs/nGREs or interacts with other transcription factors such as AP-1, NF-κB or Stat1. As a consequence, GC-responsive target genes are either activated or repressed. Phosphorylation of the GR can additionally modulate transcriptional strength. Moreover, proteins encoded by genes that are induced by GCs such as DUSP1 can act in the cytosol and repress MAPK signaling. GCs can alternatively recognize a presumed membrane-bound receptor. Finally, they can also bind to the cytosolic MR, which is only expressed in selected cell types. In this case, the ligand-bound MR translocates into the nucleus and modulates transcription predominantly by DNA-binding-dependent mechanisms.

The molecular mechanisms of the GR are manifold (Figure 1). Some effects occur very rapidly and are therefore believed to be transcription-independent [17]. These so-called non-genomic effects can be mediated through a presumed membrane-bound receptor or by interaction of the cytosolic GR with components of signaling pathways. It has been described that GCs can influence the MAPK pathway [18], T-cell receptor signaling [19], or induce a rearrangement of the cytoskeleton via phosphorylation of ERM proteins [20]. However, in most cases, the GR translocates into the nucleus after GC binding and alters transcriptional activity [21]. These genomic effects can be mediated by different modes of GR action. Recognition of palindromic DNA elements in regulatory regions that are present in many genes, designated GC responsive elements (GREs), is one option. Here, DNA-binding of GR dimers nucleates the assembly of a large transcription regulatory complex that induces gene transactivation. Alternatively, the dimeric GR can also recognize negative GREs leading to an inhibition of transcription [22], or the monomeric GR can bind to half site GREs, which is associated with both positive and negative transcriptional regulation [21,23]. It is also worth mentioning that posttranscriptional modifications of the GR such as phosphorylation can influence transcriptional strength and dictate target gene selection [24]. A mechanism which is independent of DNA-binding concerns the possibility that the GR controls gene expression by interacting with other transcription factors such as AP-1 [25], NF-κB [26], and Stat1 [27]. Historically, it was believed that such GR protein–protein interactions account for the majority of repressive GC effects, but genome-wide studies revealed that in many cases the GR rather binds to the DNA adjacent to the aforementioned transcription factors and thereby influences their activity [28,29]. Besides repressing pro-inflammatory genes, induction of anti-inflammatory factors has also been found to be decisive for immunosuppressive functions of GCs, the resolution of inflammation, and immune cell reprogramming [30,31]. Consequently, anti-inflammatory GC activities may or may not require DNA-binding of the GR [32,33].

Quite recently, GCs were found to mediate some of their anti-inflammatory activities by regulating microRNAs (miRNAs), small molecules which are known to interfere with the untranslated regions of target mRNAs and thereby fine tune gene expression [34,35]. In a mouse model of systemic inflammation, GCs induced miR-511 and thereby protected against TNFα-induced shock by reducing expression of TNF receptor 1 protein (TNFR1) [36]. Furthermore, GCs were shown to induce miR-342 in Treg cells, which is believed to make an important contribution to the anti-inflammatory activity of GCs as well [37]. Last but not least, miRNAs can impact steroidogenesis in the adrenal cortex and thereby regulate the availability of GCs [38].

As outlined below, a good understanding of the different modes of GR action, in particular the role of dimerization/oligomerization and DNA-binding, is instrumental for designing new ligands that alter DNA-recognition and thereby modify the expression profile of GC-responsive target cells.

## 3. GCs in the Pathogenesis and Treatment of Inflammatory Diseases

### 3.1. Neuroinflammatory Diseases

The central nervous system (CNS) was traditionally considered to be an immune privileged site with limited access by leukocytes and specialized features concerning tissue transplantation [39,40]. Immune ignorance is maintained by specialized barriers shielding the CNS from the circulation (blood–brain barrier and blood–liquor barrier), the absence of a lymphatic system draining the CNS parenchyma (although lymphatic vessels in the outer part of the dura mater were recently described [41,42]), and the paucity of immune factors such as signaling molecules. However, it became recently clear that a certain degree of immune surveillance in the CNS must exist to fight pathogens [40]. This consists of patrolling activated/memory T cells and the microglia, which constitute a local population of innate immune cells sharing many properties of tissue-resident macrophages [43]. In addition to these homeostatic functions, peripheral immune cells can infiltrate the CNS in response to insults such as trauma, infection, or autoimmunity following disruption of the aforementioned barriers, which then results in neuroinflammation. Since the CNS is very sensitive to damage, immune reactions need to be tightly controlled to prevent neuroinflammation and severe neurological symptoms. Endogenous GCs represent such a regulatory mechanism, which is naturally engaged during stress but can be employed as a therapeutic regimen, too.

**Figure 2:**
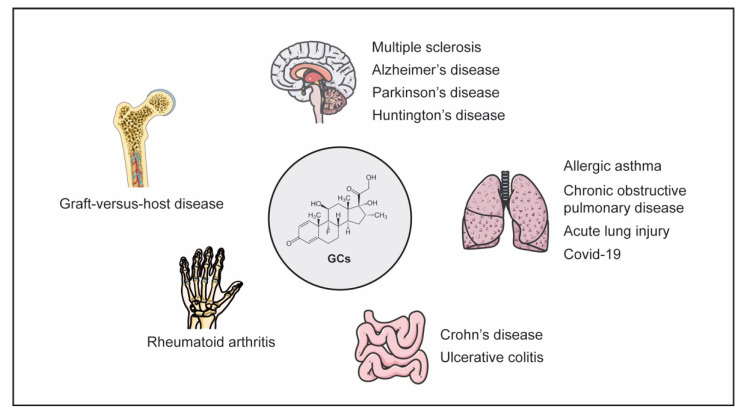
GC therapy of major inflammatory diseases. GCs are used to treat a multitude of organ-specific diseases affecting the brain or different peripheral tissues like the intestinal tract, lung or joints, but also systemic disorders such as graft-versus-host disease.

MS is a neuroinflammatory disease of autoimmune origin and is characterized by demyelination and axonal damage, eventually leading to progressive disability (Figure 2). Based on the features of the disease, relapsing–remitting MS is distinguished from primary and secondary progressive MS. High-dose intravenous methylprednisolone (MP) therapy is a mainstay in the treatment of acute relapses [44], but GCs are applied as well to patients suffering from progressive forms of MS. A widely used animal model with which to study the pathomechanisms and treatment regimens of MS is experimental autoimmune encephalomyelitis (EAE) [45]. Using this model, it was found that a wide range of GCs were able to ameliorate clinical symptoms of neuroinflammation in a dose-dependent manner [46,47,48]. An analysis of knock-out mice lacking the GR in different immune cell compartments further revealed that peripheral T cells are the major target of therapeutically applied GCs [47]. Mice harboring GR-deficient T cells developed an aggravated disease after immunization with MOG_35–55_ peptide in Complete Freund’s Adjuvant (CFA), which was refractory to Dex therapy [47]. Several studies then investigated the mechanisms by which GCs modulated T-cell function in EAE. It was found that GCs induced apoptosis in naïve T cells, while antigen-primed effector T cells were protected from cell death by expression of Bcl-2 and Bcl-X_L_ [49]. Consequently, a silencing of the GR in antigen-specific T cells had no effect on their ability to induce EAE [50]. Interestingly, the analysis of GR^dim^ mice, which express a GR with a mutation in the dimerization domain and are therefore fully resistant to GC-induced T-cell apoptosis, provided evidence that this mechanism was not essential for the ability of GCs to confer protection from severe EAE [33]. In fact, the disease course in GR^dim^ mice was similar to wild-type mice, and Dex administration was still able to ameliorate clinical symptoms of EAE. While these findings indicated that apoptosis induction was not the major mechanism of GC action in EAE, it turned out that GCs rather altered the migration of T cells along chemokine gradients and thereby prevented CNS infiltration [33]. Specifically, Dex enhanced the T cells’ responsiveness to CXCL12, and an inhibition of its receptor CXCR4 in vivo reduced the efficacy of GCs in EAE therapy. These findings indicated that GCs redirect T cell migration away from the inflamed CNS to peripheral tissues and thereby ameliorate neuroinflammation [33]. Finally, other mechanisms that also contribute to the therapeutic activity of GCs in EAE were described, including the down-regulation of cell adhesion molecules such as LFA-1 and VLA-4, which are required by T cells to cross the blood–brain barrier [47,51], and the repression of pro-inflammatory cytokines such as IFNγ, IL-17, and GM-CSF [46].

GCs also impact myeloid cells. Dexamethasone for instance was found to diminish MHC class II expression on microglia, which interfered with the re-activation of antigen-specific T cells in the CNS [52]. Accordingly, mice lacking the GR in the myeloid cell compartment developed exacerbated clinical symptoms following induction of EAE, although treatment with free GCs was still effective [47]. As outlined earlier, GCs not only bind to the GR but also to the MR, and initiate effects via both receptors in cell types such as macrophages that are devoid of 11β-HSD2 expression. Consequently, MR ablation in myeloid cells ameliorated EAE symptoms, which could be explained by a repolarization of macrophages and microglia to the M2 phenotype, thereby lowering NO production and altering phagocytic and migratory properties [53].

Collectively, preclinical data has provided compelling evidence that GCs employ various mechanisms and act on different cell types in the modulation of neuroinflammatory processes to dampen immunological processes (Table 1). This applies to endogenous GCs, in the absence of which clinical symptoms are aggravated, as well as therapeutic GC administration, which results in a mitigation of clinical symptoms [47,54].

Currently, there is an ongoing debate whether neurodegenerative disorders such as Alzheimer’s disease (AD), Parkinson’s disease (PD), and Huntington’s disease (HD) also involve inflammatory processes, which would entail therapeutic approaches that target components of the immune system (Figure 2). A generally reduced risk to develop AD was reported for individuals receiving GCs [55]. In addition, combined treatment with dexamethasone (Dex) and acyclovir resulted in diminished neuroinflammation in an AD mouse model and a concomitant protection against cognitive impairment [56]. In the case of PD, pro-inflammatory molecules secreted by microglia are believed to play a role in disease development, which could be prevented by application of synthetic GCs [57]. GR signaling in microglia promoted the neuroprotective function of GCs and prevented the loss of dopaminergic neurons in the brain following immunological challenge [58,59]. Importantly, the use of GCs in neurodegenerative diseases is not undisputed, due to their adverse effects. In an AD rat model, it was found that MP disrupted hippocampal synaptic long-term potentiation and caused cognitive dysfunction [60]. Paradoxically, pro-inflammatory activities of GCs in the CNS have been described, too, and hypothesized to be dependent on FKBP51, a protein that sequesters the GR in the cytosol [61]. Since communication between neurons and microglia is essential for maintaining neuronal homeostasis [62], a reduced microglial activity by GCs could render neurons more vulnerable to damage [63]. However, it was also found that hippocampal microglia were primed by GCs for enhancement of LPS-induced pro-inflammatory factors [64,65]. It is noteworthy that an important role of GCs in the hippocampus was already recognized long ago [66,67]. Namely, it was observed that the structural integrity of the hippocampus, memory formation, and cognition were impaired in patients after critical illness, presumably as a consequence of the activity of endogenous or exogenous GCs [68]. This was explained by enhanced ROS formation and increased expression of the inflammasome NLRP3 in hippocampal microglia after chronic stress, leading to depression-like behavior [69,70]. Generally, GCs often seem to promote the development of neurodegeneration [71,72,73], for which reason chronic stress or a dysfunctional HPA axis are considered as risk factors in AD and PD [74]. It is against this background that one has to carefully balance the pros and cons of the therapeutic use of GCs in neurodegenerative diseases.

### 3.2. Inflammatory Bowel Disease

The gastrointestinal tract is the second largest surface of the human body, and due to its multiple roles in nutrient digestion and absorption, it is constantly exposed to the external environment. Protection against harmful microorganisms is jointly achieved by intestinal epithelial cells (IECs), different types of immune cells in the lamina propria, and the microbiota which is composed of trillions of different bacteria. IECs form a physical and biochemical barrier against pathogen invasion, but they also produce inflammatory mediators and can act as antigen presenting cells (APCs) [75,76]. Immune cells such as macrophages, DCs, T cells, and IgA secretory plasma cells are highly abundant in the lamina propria and communicate through the release of cytokines and direct cell–cell contact. Noteworthy, Treg cells are also present in the gut and contribute to the control of inflammatory responses [76]. Epithelial barrier integrity and the continuous interaction between IECs, immune cells, and the microbiota are crucial to maintain gut homeostasis [77]. Hence, a dysregulation of this balance can induce inflammatory bowel disease (IBD), which is characterized by an exaggerated immune response directed against components of the digestive system. IBD includes Crohn’s disease (CD) and Ulcerative Colitis (UC) (Figure 2), which are both characterized by clinical symptoms including fever, diarrhea, abdominal pain, weight loss, nausea, and vomiting [78,79]. There is a variety of drugs that are used to treat IBD, including aminosalicylates, antibiotics, and monoclonal antibodies targeting for instance TNFα [80,81]. However, in patients with moderate to severe disease, GCs are still the standard treatment despite their various side effects. Nonetheless, some patients do not benefit from this therapy, and others at least require continuous treatment to prevent a relapse, which is referred to as steroid-dependency [82]. 

Even though GCs have been used in IBD therapy for decades, some of their effects remain poorly understood. Therefore, several animal models of colitis have been studied to define GC effects in gut inflammation (Table 2). Colitis can be either induced by dextran soldium sulfate (DSS) or dinitrobenzene sulfonic acid (DNBS) due to the components’ toxicity to epithelial cells in the colon, or by transferring T cell preparations depleted of Treg cells into immunodeficient mice [83,84]. Furthermore, intestinal inflammation can also be induced by TNFα treatment [27]. Analysis of mice with a selective deletion of the GR in myeloid cells revealed that GC activities in macrophages and neutrophils were essential for the resolution of DSS-induced colitis [83]. In the absence of the GR, macrophage abundance in the colon was increased, expression of pro-inflammatory mediators was upregulated, and CD163, CD206, and IL-10 levels were reduced. This indicates that myeloid cells adopt an M1 phenotype when the GR is absent. Analysis of DSS-induced colitis in mice carrying an inducible deletion of the GR in IECs revealed aggravated clinical symptoms already at an early stage of the disease, which was accompanied by enhanced epithelial permeability and severe inflammation in the colon [7]. Chemokine expression in IECs remained low during colitis in the absence of the GR, which impaired the recruitment of myeloid cells into the lamina propria accompanied by leukocyte hyperactivation. It is noteworthy that the exacerbated inflammation increased the susceptibility to colonic tumor development, indicating that a failure of GCs to control colitis is a risk factor for cancer. Using T cell transfer colitis, the GR in Treg cells was found to be essential for proper control of colonic inflammation as well, because it prevented them from losing their regulatory features [84]. Paradoxically, Dex administration to mice was found to exacerbate rather than ameliorate DSS-induced colitis [85], which was confirmed in a study using liposomal Dex [86]. The experimental model therefore needs to be chosen with care.

A typical feature of IBD is the compromised epithelial barrier function in the colon, which is mediated amongst others by TNFα that reduces the expression of tight junction proteins [87]. Consequently, stimulation of organoids from CD patients with a cocktail of pro-inflammatory cytokines increased STAT1 phosphorylation, altered the expression of E-cadherin, Claudin-2, and MLCK, and enhanced epithelial permeability [88]. All these effects could be counteracted by prednisolone application, providing an explanation for the therapeutic mechanism of GCs in IBD. In line with these results, GR^dim^ mice were found to be prone to TNFα-induced intestinal inflammation and the concurrent loss of epithelial barrier integrity, which was explained by the observation that the monomeric GR was unable to block STAT1 activity in IECs [27].

Some recent work focused on the role of GILZ in IBD, an anti-inflammatory gene induced by GCs [89]. The DNBS-induced colitis model was used to study effects of GILZ deficiency in neutrophils. These mice developed aggravated clinical symptoms due to the neutrophils’ activated phenotype, which was explained by a failure of GCs to inhibit the MAPK pathway [90]. Analysis of mice lacking GILZ specifically in T cells revealed an impaired Treg cell differentiation, which also resulted in aggravated clinical symptoms in DNBS-induced colitis [91]. Importantly, this phenotype is well in line with the finding that GR-deficient Treg cells fail to control T cell transfer colitis [84]. There is also evidence that induction of FoxP3 by GCs and enhanced Treg cell differentiation require cooperation with TGF-β. Hence, the role of TGF-β1 was also investigated in a murine model of colitis. Low amounts of TGF-β1 directly delivered into the colon reduced colonic inflammation and restored Treg cell numbers. Dex supported the differentiation of Treg cells in the presence of TGF-β1 and alleviated clinical symptoms, indicating that the activity of GCs in the treatment of colitis may depend on an interaction of the GR with components of TGF-β1 signaling [92].

**Table 2:** Phenotype of selected genetic mouse models of IBD. n.d.: not determined.

Gene	Type of Mutation	Model	Disease	Therapy (Free GCs)	Reference
GR	myeloid cell-specific deletion	DSS-colitis	aggravated	n.d.	[83]
GR	IEC-specific deletion, inducible	DSS-colitis	aggravated	n.d.	[7]
GR	Treg cell-specific deletion	T-cell transfer colitis	aggravated	n.d.	[84]
GR	impaired dimerization, ubiquitous	TNFα-induced intestinal inflammation	aggravated	abrogated	[27]
GILZ	neutrophil-specific deletion	DNBS-colitis	aggravated	n.d.	[90]
GILZ	T cell-specific deletion	DNBS-colitis	aggravated	n.d.	[91]

Dysbiosis is common during IBD. Some GCs including Dex increase the abundance of *Bifidobacterium* and *Lactobacilli* while others have no effect on the composition of the microbiota [93]. The clinical relevance of this observation, however, remains unknown. In contrast, an established option to influence the gut microbiome is *exclusive enteral nutrition* (EEN), a common first-line treatment of CD in children, which involves the application of a nutrition formular drink and the exclusion of solid food. A clinical trial comparing the efficacy of GCs and EEN revealed that both treatments induced remission although EEN was superior in inducing mucosal healing while causing less adverse effects [94]. Another option to replace GCs in the treatment of IBD is *fecal microbiota transplantation* (FMT). A study of steroid-dependent UC patients indicated that FMT is indeed a promising strategy to achieve clinical remission with little side effects and can help with GC withdrawal [95].

### 3.3. Pulmonary Diseases

Similar to the gastrointestinal tract, the lung is constantly exposed to environmental threats such as microorganisms, allergens, and chemical substances [96,97]. The airway epithelium forms a protective physical barrier that is sustained by tight junctions, but it is also involved in maintaining immune homeostasis by secreting a variety of cytokines and chemokines, expressing pattern-recognition receptors, and acting as APCs. Furthermore, multiple cell types of the innate and adaptive immune systems are found in the lower respiratory tract, including macrophages, DCs, and mast cells, which help to eliminate pathogens. However, it can also happen that the immune system mistakenly responds to intrinsically harmless antigens such as grass pollen, resulting in allergic reaction, or that the immune system responds to foreign pathogens with inappropriately great strength as is the case in COVID-19. In both situations, the dysregulated immune system leads to the development of severe inflammatory lung diseases (Figure 2).

Asthma and chronic obstructive pulmonary disease (COPD) are the most frequent respiratory disorders and affect more than 500 million people worldwide [98]. Asthma is characterized by a hyperresponsiveness of the airways to environmental factors and can cause symptoms such as cough, dyspnea, or wheezing [99]. It is a heterogeneous disorder that is subdivided in several categories according to its phenotype and immune profile, for instance based on the predominance of either eosinophils or neutrophils [100,101]. Most patients suffer from the allergic variant of the disease, which is triggered by an adaptive immune response against inhaled antigens. After antigen recognition, DCs migrate to the lymph nodes, where they activate naive CD4^+^ T cells and induce their differentiation into Th2 cells [102]. Allergic asthma is characterized by high serum levels of IgE, infiltration of eosinophils into the lung, degranulation of mast cells, and the release of inflammatory cytokines such as IL-4, IL-5, and IL-13 [99,102]. The neutrophilic variant of asthma has a different pathomechanism and mainly involves Th17 cells. Expression of pro-neutrophilic factors such as IL-8, IL-1β, and IL-6 are increased in this subtype and suspected to relate to subclinical infections since neutrophilic infiltration is tightly linked to LPS exposure [99,102]. In contrast to asthma, COPD is not an antigen-driven disease but rather caused by an inflammatory response to inhaled irritants such as tobacco smoke, which results in a remodeling of the airways accompanied by limited airflow, an emphysema, and chronic bronchitis [103]. GCs are used in the treatment of all types of inflammatory lung disorders although with different efficacy. Whereas GCs are employed as standard care of allergic asthma patients, [104], they are much less effective in neutrophilic asthma. COPD patients respond poorly to GCs, too, but there is still an ongoing debate concerning the true benefit of this therapy [105,106]. The responsiveness of inflammatory lung diseases to GCs seems to be linked to their pathomechanism: they induce apoptosis in eosinophils but they inhibit the same in neutrophils [107,108]. This observation provides a plausible explanation why neutrophilic asthma and COPD are refractory to GCs while allergic asthma responds well. Noteworthy, allergic asthma patients can also be resistant to GC therapy, but the mechanisms are manifold and poorly understood. For example, GR activity can be reduced by phosphorylation via p38 [109], which compromises the ability of the GR to translocate into the nucleus and regulate transcription [110].

Many insights into the mechanisms of GCs in allergic asthma have been obtained from mouse models. Surprisingly, the GR in immune cells was found to be dispensable for treatment of allergic airway inflammation induced by immunization with ovalbumin adsorped to aluminum hydroxide, which represents a Th2-dependent model of asthma (Table 3). In contrast, GR expression in non-hematopoietic stromal cells in the lung, in particular alveolar epithelial type 2 (AT2) cells, was crucial for successful GC therapy [8]. Furthermore, a GR with an intact dimerization interface was required to resolve airway inflammation and hyperresponsiveness through its ability to modulate transcription of genes predominantly expressed by airway epithelial cells. To clarify the mechanism underlying the refractoriness of neutrophilic asthma to GC therapy, a Th1-dependent model induced by immunization with ovalbumin in CFA was used. Interestingly, it was found that GC responsiveness in this model could be restored by blocking TNFα [111]. Collectively, these studies entail new approaches for asthma therapy based on selectively targeting airway epithelial cells or using TNFα inhibitors.

ALI and its most severe form, acute respiratory distress syndrome (ARDS), are life-threatening inflammatory disorders characterized by different degrees of hypoxemia, pulmonary edema, and leukocyte infiltration, and can result from severe trauma or sepsis [112,113]. The mortality rate of ALI is extremely high and can exceed 40% [114,115]. Pathophysiological hallmarks are a disruption of endothelial–epithelial barrier integrity in the alveoli and increased production of pro-inflammatory cytokines and chemokines, which promote leukocyte infiltration, especially of neutrophils [116,117]. Treatment of ALI is mostly based on supportive care including mechanical ventilation, but application of GCs is also an option. Although a meta-analysis provided evidence that the use of GCs was recommended in ALI therapy [118], their true efficacy still remains debatable [119]. Furthermore, potential benefits of GCs, including the suppression of inflammation and proliferation, have to be balanced with their adverse effects and the increased risk of infection [119]. In several clinical trials, the use of a low dose of GCs in the early phase of ARDS/ALI was effective and reduced mortality. In contrast, patients did not benefit from GC treatment when performed at later stages but rather experienced side effects [120,121]. Nevertheless, clinical trials were overall inconclusive and clear recommendations for the treatment of ALI with GCs are still missing.

To obtain a better understanding of the mechanisms of GCs in ALI therapy, animal experiments were conducted. In a mouse model of ALI induced by LPS injection, MP treatment ameliorated inflammation, improved lung function, and reduced tissue injury. The beneficial effects of GCs could be linked to the polarization of macrophages towards the M2 phenotype and an induction of Treg cells [122]. In another study, an ALI model based on the combined application of LPS and oleic acid was used. Dex efficiently reduced lung inflammation and restored epithelial barrier integrity, effects that were mediated by the dimeric GR in myeloid cells (Table 3). The therapeutic GC activity depended on an upregulation of SphK1 in macrophages, resulting in elevated S1P serum levels. Since S1P is a major regulator of the endothelial permeability in the lung, its induction by GCs is instrumental in ameliorating ALI [123]. Finally, GR^dim^ mice showed a reduced survival in LPS induced inflammation under intensive care treatment, accompanied by impaired lung mechanics and gas exchange, indicating that endogenous GC actions mediated via the dimeric GR are crucial for circulatory and pulmonary functionality [124].

**Table 3:** Phenotype of selected genetic mouse models of pulmonary diseases. Ova/Alum immunization: allergic airway inflammation induced by immunization with ovalbumin adsorped to aluminum hydroxide. LPS/Oleic acid treatment: chemically induced model of ALI.

Gene	Type of Mutation	Model	Disease	Therapy (Free GCs)	Reference
GR	deletion in immune cells	Ova/Alum immunization	unaltered	unaltered	[8]
GR	deletion in stromal cells	Ova/Alum immunization	unaltered	abrogated	[8]
GR	impaired dimerization, ubiquitous	Ova/Alum immunization	unaltered	abrogated	[8]
GR	AT2-specific deletion, inducible	Ova/Alum immunization	unaltered	partially abrogated	[8]
GR	myeloid cell-specific deletion	LPS/Oleic acidtreatment	unaltered	abrogated	[123]
GR	impaired dimerization, ubiquitous	LPS/Oleic acid treatment	unaltered	abrogated	[123]

COVID-19 is a life-threatening disease caused by an infection with the SARS-CoV-2 virus, which has recently emerged all over the world. It has been associated with a high mortality rate caused by ARDS/ALI, raising the question whether GCs possibly are a suitable therapy for handling the cytokine storm in severely affected patients [125]. At the very beginning of the pandemic, this treatment regimen was regarded with some caution, considering the immunosuppressive activity of these drugs. However, a retrospective analysis of nine COVID-19 patients from Wuhan in China performed early in 2020 provided evidence that a medium to low dose of MP improved both inflammation and clinical parameters [126]. Several clinical trials conducted during the last 18 months then provided further convincing evidence that GC administration was beneficial in COVID-19-associated ARDS. In the RECOVERY study, which is the largest clinical trial up to now, almost 10,000 patients were analyzed. A lower 28-day mortality rate was observed after administration of Dex to patients receiving invasive mechanical ventilation or oxygen supplementation alone. In contrast, patients without respiratory support did not significantly benefit from GC therapy [127]. Results from another clinical trial recommended the application of high-dose MP, followed by tocolizumab, because it was observed that this therapeutic regimen reduced mortality and accelerated respiratory recovery [128]. A meta-analysis confirmed the effectiveness of GCs in the treatment of critical-ill COVID-19 patients, but the optimal time point and drug dose remains debatable [128,129]. Albeit MP improves the disease [130], it was also shown that this drug not only suppresses cytokine production but also boosts viral RNA replication. Pulmonary inflammation was alleviated by GCs in a hamster model of SARS-CoV-2, but the viral load was concomitantly increased and antibody levels against the spike protein were reduced [131]. It is noteworthy that the negative effects of GCs could be prevented by additionally applying remdesivir, which may inspire clinical trials addressing the efficacy of a combinational therapy with both drugs [131]. Although further work is required to define the optimal treatment regimen, the WHO recommends the use of GCs in the management of severe cases of COVID-19-associated ARDS without restriction [132].

### 3.4. Rheumatoid Arthritis

GCs have been successfully used to treat RA patients since 1948 [133], a milestone of medical history which was awarded the Nobel prize only two years later. RA is an autoimmune disease that affects 0.5–1% of the population worldwide (Figure 2) [134,135,136], in particular elderly women [137]. Genetic risk factors such as the HLA haplotype, especially variations of the HLA-DRB1 gene, health conditions including smoking, obesity, and cardiovascular diseases, as well as environmental factors are known to contribute to the pathogenesis of RA [138]. Major hallmarks comprise a persistent synovial inflammation resulting in joint damage as well as cartilage and bone destruction. These pathological features are caused by rheumatoid factors, anti-citrullinated peptide antibodies, and pro-inflammatory mediators that activate the innate immune system. In addition, T and B cells become primed and then initiate the production of autoantibodies [139]. In combination, these innate and adaptive immune processes eventually lead to an activation of synovial fibroblasts and macrophages through the release of cytokines and cause an exacerbated inflammatory response. There is a plethora of medications available to treat RA such as non-steroidal anti-inflammatory drugs (NSAID), disease-modifying anti-rheumatic drugs (DMARDS), and biologicals such cytokine-neutralizing monoclonal antibodies, which cause a quick relief from clinical symptoms, but GCs remain indispensable for the management of RA even today due to their unsurpassed efficacy.

Crucial insights into the mechanisms of GCs in RA were obtained in animal models. Each model reflects different phases of the inflammatory response and also the involved cell types. The TNFα-transgenic mouse model includes multiple cell types, the antigen-induced arthritis (AIA) and the glucose-6-phosphate isomerase (G6PI) model are both T cell-dependent, and the collagen-induced arthritis (CIA) and the serum-transfer induced arthritis (STIA) model require macrophages and mast cells but are independent of T-cell functions [140].

Analysis of conditional GR knockout mice using experimental models of arthritis revealed how GC-mediated anti-inflammatory effects were regulated through the GR in distinct cell types and tissues, sometimes leading to surprising discoveries such as GC effects in non-hematopoietic cells. In addition, mice were analyzed that lack 11β-HSD1, leading to a reduced conversion of inactive GCs like cortisone and deoxycorticosterone to their active counterparts, cortisol and corticosterone, as well as mice that are deficient for GC target genes such as GILZ (Table 4). Using this approach, the GR in T cells was found to be crucial for immune suppression by GC treatment in the AIA model, which partially depends on Th17 cells [141]. In contrast, the GR in other cell types was dispensable for GC action in this model. Namely, clinical symptoms could be repressed in mice with conditional deletions of the GR in macrophages, DCs, or B cells, indicating that the GR in these cells was not essential for the anti-inflammatory effects of GCs [141]. Analyses performed in the STIA model that is independent of adaptive immunity, revealed that non-classical monocytes were essential for the initiation of arthritis and give rise to inflammatory macrophages during disease progression as shown by depletion experiments [142]. Surprisingly, GR deletion in all hematopoietic cells or specifically in macrophages failed to abrogate the anti-inflammatory effects by GCs in the STIA model [143]. In contrast, the GR dimer in stromal cells was essential for the anti-inflammatory activity of GCs and their clinical efficacy [143]. This observation was explained by the fact that GCs normally induce alternative macrophages during arthritis, which is no longer possible in the absence of the stromal GR. Interestingly, mice lacking the GR specifically in fibroblast-like synoviocytes (FLS) were at least partially resistant to GC-induced repression in the STIA model. Here, evidence was obtained that an inadequate clearance of apoptotic cells after GC therapy and impaired macrophage polarization caused persistent inflammation. The importance of the cross-talk between FLS and macrophage is further emphasized by studies with specific deletions of 11β-HSD1 in these two cell types, which suggest that GCs need to be locally activated in macrophages before being able to reduce inflammation [144]. The fine tuning of this cross-talk between different kinds of cells by GCs and the discovery that FLS exist in different pro-inflammatory subtypes [145] require further investigation which entails answering the intriguing question of how GCs impact on these subtypes to control inflammation during therapy.

The role of endogenous GCs in RA was addressed using 11β-HSD1-deficient mice. In models like STIA and TNF-α induced arthritis, they showed an exacerbated inflammation, a retarded resolution of the immune response, increased joint destruction, as well as enhanced bone loss [146,147]. In contrast, chemical inhibition of 11β-HSD1 activity in the CIA model reduced synovial inflammation, joint destruction, and serum levels of inflammatory cytokines [148], indicating different outcomes after either deleting or blocking 11β-HSD1. Interestingly, endogenous GCs seem to have dual effects in RA. Anti-inflammatory activities are established in macrophages, mast cells, and chondrocytes, whereas GCs exert pro-inflammatory effects in osteoblasts, myocytes, and FLS [149]. Mice overexpressing the GC-inactivating enzyme 11β-HSD2 in osteoblasts showed an attenuated local inflammatory activity and clinical signs of arthritis [150], whereas mice carrying a specific deletion of the GR in chondrocytes developed exaggerated clinical symptoms in the STIA and AIA models. The latter finding thus provides evidence that GCs acting via the GR in chondrocytes reduce inflammation [151].

In contrast to the endogenous hormones, GCs applied in a therapeutic setting exert anti-inflammatory effects in preclinical models similar to patients. GR^dim^ mice were refractory to Dex treatment in models of AIA, G6PI and STIA, and showed an increased clinical score and aggravated ankle swelling regardless of Dex application [141,143]. It is known that major mediators of anti-inflammatory GC effects are encoded by GR dimerization-dependent target genes such as Annexin A1, GILZ, and Dusp1 [3]. The anti-inflammatory molecule Annexin A1 is induced by GCs in monocytes and neutrophils [152,153] and mediates diverse effects in adaptive and innate immunity, for which reason it is believed to be an important player in RA models [154,155]. Thus, it is required to reduce inflammation in the STIA model, whereas Annexin A1-deficient mice are resistant to GC treatment [156]. Annexin A1 limits leukocyte recruitment, induces apoptosis of neutrophils, and enhances efferocytosis of macrophages [157,158,159,160]. GILZ is also induced by GCs and modulates inflammatory responses through its interaction with NF-κB, AP-1, and other transcription factors [89]. Mice in which CIA was induced as well as patients suffering from RA have enhanced GILZ levels in the synovium after GC treatment, providing evidence for an anti-inflammatory role of GILZ in arthritis [161]. Surprisingly, GILZ-deficient mice respond to exogenous GC treatment in the CIA model, whereas inflammation in the same model is attenuated in mice overexpressing GILZ in the joints [162]. These studies collectively endorse a role of GILZ as anti-inflammatory GC-induced protein with therapeutic potential. Dusp1 is also upregulated by GCs via the dimerized GR and contributes to the resolution of inflammation [18]. Dusp1 knockout mice show an exacerbated disease course and an earlier onset in the CIA model, along with increased osteoclast numbers in the joints and enhanced bone loss [163]. Synovial biopsies obtained from RA patients revealed a strong downregulation of Dusp1 [164], supporting the notion that Dusp-1 deficiency contributes to RA progression.

**Table 4:** Phenotype of selected genetic mouse models of RA. AnX1: Annexin A1. n.d.: not determined.

Gene	Type of Mutation	Model	Disease	Therapy (Free GCs)	Reference
GR	T cell-specific deletion	AIA	unaltered	abrogated	[141]
GR	myeloid cell-specific deletion	AIA	unaltered	unaltered	[141]
GR	B cell-specific deletion	AIA	unaltered	unaltered	[141]
GR	impaired dimerization, ubiquitous	AIA	unaltered	abrogated	[141]
GR	deletion in immune cells	STIA	unaltered	unaltered	[143]
GR	deletion in stromal cells	STIA	unaltered	abrogated	[143]
GR	FLS-specific deletion, inducible	STIA	unaltered	partially abrogated	[143]
11β-HSD1	ubiquitous deletion	STIA	aggravated	partially abrogated	[144,147]
AnX1	ubiquitous deletion	STIA	unaltered	abrogated	[156]
GILZ	ubiquitous deletion	CIA	unaltered	unaltered	[162]
DUSP1	ubiquitous deletion	CIA	aggravated	n.d.	[163]

### 3.5. Graft-Versus-Host Disease

Hematopoietic stem cell transplantation (HSCT) often remains the only curative approach to treat myeloid and lymphoid malignancies [165]. In the case of allogeneic HSCT, pluripotent stem cells from the bone marrow or peripheral blood of an unrelated donor are transferred to a preconditioned patient and there support the reconstitution of a new and healthy immune system. The T cells contained in the graft contribute to the success of this therapy since they help to eliminate residual malignant cells which remain in the patient after the conditioning regimen [166]. This beneficial activity is termed graft-versus-leukemia (GvL) effect. Unfortunately, the allogenic T cells are also responsible for the most severe complication of HSCT, namely graft-versus-host disease (GvHD) (Figure 2). After recognizing unmatched MHC and other molecules in the recipient, they become activated and subsequently induce severe tissue damage, in particular in skin, liver, and the intestinal tract [167].

The acute form of GvHD (aGvHD), which develops in about half of all patients, is a major risk factor for morbidity and mortality in the early post-transplantation phase. A frequently employed strategy to prevent aGvHD is the inactivation of allogeneic T cells with the immunosuppressive drugs cyclosporin A and methotrexate. In contrast, therapy of aGvHD is generally achieved by intravenous administration of prednisone [168]. This strategy, however, is complicated by side effects of GCs and an increased risk of infections caused by viruses, encapsulated bacteria, and fungi [169]. Strong efforts are therefore being made to improve the features of GC therapy. A recent phase I trial compared the use of prednisone and the mTOR inhibitor sirolimus in the first-line therapy of aGvHD. Treatment efficacy of sirolimus was moderately lower compared to prednisone but the side effects were considerably less, which was accompanied by an increased quality of life [170]. In another recent clinical trial, it was observed that progression of moderate disease was efficiently diminished by GCs, while further development of severe aGvHD could not be prevented [171]. This finding entails the use of GCs immediately after diagnosis rather than at advanced stages. Identification of plasma biomarkers that would allow predicting the outcome of aGvHD are an alternative approach to improve GC therapy but up to now the results have been disappointing [172]. In cases where no satisfactory improvement of the disease is achieved with GCs, second-line therapies based on the application of monoclonal antibodies or extracorporal photopheresis have to be initiated [173,174].

GCs constitute a well-established therapy of aGvHD but only very few studies have been reported addressing their cellular and molecular mechanisms. In a haploidentical HSCT mouse model, GC treatment ameliorated aGvHD when initiated at an early stage of the disease. This clinical effect was accompanied by a reduced expression of cytokines, chemokines, and adhesion molecules in the gastrointestinal tract but to a lesser extent in liver [175]. In contrast, GC administration at an advanced stage of aGvHD was ineffective, which is in line with the corresponding clinical observation [171]. The roles of different immune cell subpopulations in GC therapy were deciphered in a fully MHC-mismatched aGvHD mouse model (Table 5). HSCT involving GR-deficient T cells resulted in a devastating disease course characterized by severe diarrhea, a strong loss of body weight, hypoglycemia, and hypothermia as well as considerable tissue damage in the small intestine [176]. These effects were accompanied by a pronounced infiltration of CD8^+^ T cells into target organs and an enhanced cytotoxic T-cell activity. Using a single MHC class I-disparate aGvHD model, the importance of CD8^+^ T cells as targets of GCs in aGvHD were confirmed [176]. A possible strategy to reduce the risk of infections after HSCT is the adoptive transfer of virus-specific T cells. This strategy, however, is compromised by the fact that GCs applied to aGvHD patients induce apoptosis also in the virus-specific T cells. Thus, effort has been made to inactivate the GR in these cells by genetic manipulation, which allowed them to escape cell-death and retain antiviral activity [177,178]. Interestingly, modulation of myeloid cells by GCs plays an essential role in aGvHD as well. HSCT into mice carrying a macrophage-specific deletion of the GR caused severe clinical symptoms and early lethality [179]. Surprisingly, the aggravated disease in this model was unrelated to tissue damage but rather associated with high levels of pro-inflammatory mediators such as IL-6. It therefore appears that a failure of endogenous GCs to control macrophage function results in a lethal cytokine release syndrome after the onset of aGvHD [179]. Finally, HSCT into mice ubiquitously expressing a dimerization-impaired GR also resulted in a fulminant disease, indicating that a fully functional GR in recipient cells is essential to control aGvHD [179].

A first step towards the optimization of GC therapy was a large-scale expression analysis performed in a fully mismatched aGvHD mouse model, which led to the discovery of a variety of genes altered in GC-resistant aGvHD. The encoded proteins could be classified as cytokines, chemokines, cell surface receptors, and intracellular molecules, and were expressed by T cells, macrophages, and non-hematopoietic cell types [180]. Although the contribution of each of these proteins remains partially unknown, they represent promising targets for future therapies. A particularly interesting group of genes is involved in the control of energy metabolism. It has been previously observed that repression of glucose transport in allogeneic T cells compromised their ability to induce aGvHD [181]. Furthermore, metabolic reprogramming was found to enhance the GvL activity of allogeneic T cells both in mice and humans [182]. It is thus conceivable that the newly identified GC-responsive metabolic genes such as glucose transporter 1 and 3 will entail new therapeutic approaches targeting this pathological mechanism [180].

## 4. Novel Therapeutic Approaches

**Figure 3:**
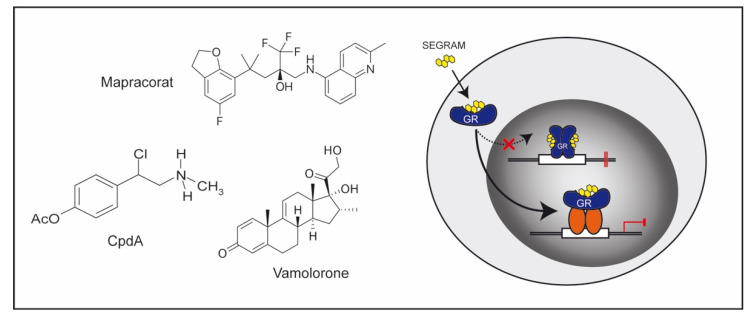
GC derivatives acting as SEGRAMs. Steroidal and non-steroidal compounds such as Mapracorat, Vamolorone, and CpdA have been developed that bind to the GR but still dissociate its molecular mechanisms based on their impaired ability to induce GR dimerization and DNA-binding. Hereby, gene transactivation and transrepression are altered, resulting in a different gene expression profile associated with an improved therapeutic index.

**Figure 4:**
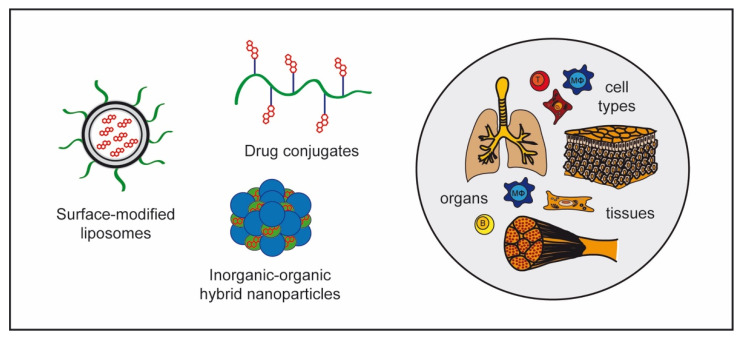
GC nanoformulations. Encapsulation of GCs in nanostructures of diverse chemical nature makes it possible to selectively target individual cell types, tissues, and organs, thus reducing systemic side effects of the drug. These novel nanoformulations include surface-modified liposomes, inorganic-organic hybrid nanoparticles, and different drug conjugates.

GCs are an important class of anti-inflammatory drugs, but adverse effects can occasionally outbalance their therapeutic benefits. Two major strategies are therefore being used to reduce side effects (Figure 3 and Figure 4). GC derivatives with a tailored mode of action can bind to the GR but affect expression of only a subset of target genes. Such compounds have been designated *Selective GR Agonists and Modulators* (SEGRAMs) [11]. In addition, novel pharmacological formulations have been developed that allow GCs to be selectively delivered to individual cell types, a strategy that aims to reduce the effective dose of the drug and avoid exposure of uninvolved cell types [12,183,184]. Both approaches have been tested in preclinical models and first examples of successful transfer into clinical application have been reported. Alternatively, it is also possible to modify GC action by targeting 11β-HSD1 [185]. Mice with a ubiquitous deletion of this enzyme were found to be largely resistant to GC treatment in experimental models of arthritis (Table 4), and showed severe joint destruction, synovitis, and leukocyte infiltrations [144]. Clinical trials testing the application of selective 11β-HSD1 inhibitors, however, have been unsuccessful up to now [15,186].

### 4.1. GC Derivatives

The GR can act via different modes of action as elaborated above [3]. Historically, GR transactivation was believed to mostly involve DNA-binding-dependent mechanisms and account for many adverse effects. In contrast, transrepression was assumed to be predominantly mediated by GR protein–protein interactions and underlie many of the beneficial anti-inflammatory GC activities. This concept was originally derived from in vitro observations showing that GR point mutations were able to dissociate these two prototypic modes of action [187,188]. Subsequently, GR^dim^ mice were developed to study these activities in vivo [189]. Due to the introduction of the A458T mutation, the GR in these mice is impaired in GRE-dependent transcriptional regulation while being fully competent of suppressing NF-κB and AP-1-dependent genes in T cells, macrophages, and fibroblasts [190]. Notwithstanding the attractiveness of this model, it turned out that the situation was more complex. GC suppression of contact allergy, arthritis, and allergic asthma was abrogated in GR^dim^ mice, while this was not the case for the therapeutic effect of GCs in EAE [8,33,141,191]. A similar dichotomy was observed for adverse effects. Hyperglycemia was no longer observed in GR^dim^ mice after GC treatment, while osteoporosis and muscle atrophy still developed [192,193,194]. These findings indicate that neither the beneficial nor the deleterious GC activities can be unequivocally assigned to one or the other molecular mechanism of the GR. A possible explanation for the ambiguous situation in GR^dim^ mice is provided by the recent observation that the A458T mutation disrupts only one of the GR dimerization interfaces thus allowing some residual binding as dimers [195]. Otherwise, GR^dim^ mice undoubtedly show a reduced transcriptional response [192], suggesting that a partial impairment of GR activity is sufficient to blunt anti-inflammatory GC effects [196]. Although these results collectively indicate that individual GR activities may be difficult to dissociate, GR^dim^ mice still encouraged basic scientists and pharmaceutical companies to screen for new compounds with a favorable therapeutic index resulting in at least a partial dissociation of their activities. These efforts resulted in the development of SEGRAMs (Figure 3).

RU24858 is the first compound that was reported to repress AP-1 mediated genes such as IL-1β without transactivating the GR, thus qualifying it as a bona fide SEGRAM [197]. This steroidal drug then became the prototype of dissociating GR ligands, although many subsequently developed SEGRAMs were non-steroidal molecules. Over the last 20 years, plenty of SEGRAMs have been characterized and tested in preclinical models [11]. AL-438, for instance, exhibited potent anti-inflammatory activity, whereas the deleterious effects on bone and glucose metabolism otherwise observed for traditional GCs were diminished [198]. A similar observation was made for ORG 214007-0, a SEGRAM that repressed CIA in mice but induced neither hyperglycemia nor muscle wasting [199]. Mapracorat (ZK 245186 or BOL-303242X) is a clinically advanced SEGRAM, which is currently being tested for the management of eye diseases [200,201]. Several clinical studies including a phase 3 trial investigating the treatment of ocular inflammation after cataract surgery (NCT01298752) were conducted, but the final results have not been published yet. One of the earliest SEGRAMs is CpdA, a synthetic analogue of a natural substance isolated from an African shrub. CpdA shows anti-inflammatory activity in mouse models of autoimmune neuritis, RA, EAE, and asthma [202,203,204,205,206,207] without inducing diabetes, skin atrophy, and osteoporosis [202,208,209,210]. Despite these favorable features, CpdA was found to be toxic when applied at high dosages to mice, which poses a potentially serious health risk for human patients [204].

VBP15, with the generic name vamorolone, is a promising example of a SEGRAM with proven clinical benefit. It was selected as the lead compound from a series of dissociating Δ9,11 GC analogues that lack GRE-mediated transcriptional activity while inhibiting pro-inflammatory NF-κB signaling [211]. VP15 has been tested in a number of preclinical models over the last couple of years and was found to reduce lung inflammation and clinical symptoms of colitis in mice while sparing adverse effects of traditional GCs [212,213]. Most importantly, treatment with VBP15 was accompanied by reduced muscle atrophy and body weight loss compared to prednisolone, which qualified this compound for application in children [214]. A rare disease affecting young boys that is widely treated with GCs is Duchenne muscular dystrophy (DMD). In fact, daily prednisone is considered to be the standard of care in some countries and shows efficacy in slowing down deterioration of muscle function. However, side effects such as growth retardation, bone fragility, and mood changes in the young patients called this therapy into question [215,216]. These complications entailed efforts to test VBP15 for the treatment of DMD. A phase 1 trial (NCT02415439) as well as a phase 2a trial (NCT02760277) were recently completed and confirmed that this novel SEGRAM was well tolerated and safe. Biomarker studies further indicated a loss of traditional GC side effects including insulin resistance, bone fragility, and adrenal suppression [217,218]. An open label study provided evidence that VBP15 improved muscle function within the 24-week observation period with minimal adverse effects [219]. A phase 2b trial intended as an admission study is currently running to assess the improvement of motoric function in young boys (NCT03439670). Notably, vamorolone has recently qualified for orphan drug designation by the FDA and EMA, a condition that indicates that it can be used to treat very rare life-threatening diseases such as DMD for which no satisfactory treatment exists. Vamorolone is therefore the clinically most advanced SEGRAM up to now.

### 4.2. GC Nanoformulations

Although a plethora of nanoformulations have been developed to improve GC delivery, most attention has been paid to pegylated or glutathione-coated liposomes, which have an increased half-life in vivo and allow the selective delivery of cargo to certain cell types (Figure 4). The pharmacokinetics and therapeutic efficacy of such modified liposomes containing Dex, MP, or prednisolone, were superior when compared to the respective free drug in EAE and preclinical models of stroke [46,220,221,222,223,224,225]. In contrast to free GCs, which mostly act on T cells, liposome-encapsulated GCs also target myeloid cells and cause a shift from an M1 to an M2 phenotype [46], an effect that could be confirmed using Dex-loaded liposomes [86]. Whereas GC treatment with liposomes was beneficial in EAE models, it worsened disease severity in DSS-induced colitis, highlighting that the success of such a strategy may differ between inflammatory diseases [86]. A new approach is the development of folate-conjugated double-layered liposomes where the drug is encapsulated within the inner membrane, with folate being attached to the outer layer [226]. Hereby, liposomes can be targeted to the folate receptor expressed on activated macrophages, which resulted in a ten-fold higher accumulation of the encapsulated drug in the inflamed joint in a rat model of RA, accompanied by an enhanced amelioration of clinical symptoms [226]. Following the same strategy, sialic acid-modified liposomes containing Dex [227] as well as hyaluronic acid-coated liposomes combining Dex and diclofenac [228] have been used to treat RA. To improve aGvHD treatment, a Dex–palmitate emulsion has been applied to mice and found superior to free GCs in clinical efficacy, which was attributed to the preferential uptake of this nanoformulation by macrophages [229].

As an alternative to lipid-based nanoformulations, novel inorganic–organic hybrid nanoparticles (IOH-NP) have been developed to carry GCs (Figure 4). They are composed of an inorganic cation and a functional organic GC anion, which are assembled into particles in a similar manner as sodium chloride [230]. IOH-NPs are almost exclusively taken up by myeloid cells and show a highly selective organ distribution after in vivo application in mice [231]. IOH-NPs containing betamethasone were employed in preclinical models of MS [48], allergic asthma, and paw inflammation [232]. In addition, GC-containing IOH-NPs were tested in a mouse model of aGvHD, where they successfully mitigated disease symptoms while partially retaining the beneficial GvL effect of the graft [233]. More specifically, the development of an adoptive B-cell lymphoma was delayed when GCs were applied using IOH-NPs instead of in their free form. This novel nanoformulation therefore has the potential to reduce the risk of relapse after HSCT, which often occurs after traditional GC therapy.

Besides classical liposomes and IOH-NPs, several other NP formulations have been designed (Figure 4). Poly-δ-decalactone/methoxy-polyethylene glycol-based NPs loaded with triamcinolone were superior to the free drug in a model of RA [234], and the use of cyclodextrin-based NPs loaded with Dex turned out to be highly efficient in the AIA model combined with reduced side effects [235]. The use of cyclodextrin improved the stability and concurrent bioavailability [236], and caused enhanced extravasation at the site of inflammation via the EPR effect [235]. An accumulation of the drug in inflamed joints, a better therapeutic efficacy, and milder adverse effects could be achieved when pH-sensitive acetone-based ketal-linked prodrugs of Dex were formulated as NPs [237]. To deliver Dex into the cartilage, poly-β-amino-ester drug conjugates of Dex were synthesized, which resulted in an enhanced uptake and reduced cartilage degradation [238]. Another recent development are biomimetic exosomes with a surface modification of folic acid-polyethylene glycol-cholesterol leading to a slow, sustained drug release [239]. Compared to other nanoformulations, the modified exosomes containing Dex showed the greatest accumulation in joints at every time point and were beneficial in a murine RA model [239]. Efforts were also made to improve GC therapy of IBD. The encapsulation of Dex into PEGylated rosmarinic acid-derived NPs was highly efficient in the treatment of DSS-induced colitis [240]. Oral administration of chitosan-modified lipid NPs made it possible to specifically target the colon where the encapsulated GCs became released due to the esterase-responsive properties of the carrier and improved experimental colitis in mice [241]. Eudragit^®^ S100-aminoclay double-coated liposomes containing budesonide were developed as pH-sensitive drug carriers for oral application. Importantly, these NPs release only low amounts of GCs in gastric fluids while the drug is efficiently mobilized at the higher pH present in intestinal fluids [242]. Finally, rectal treatment was tested as an alternative delivery route using budesonide in hydroxypropyl-beta-cyclodextrin incorporated into a thermoreversible hydrogel [243]. Whether any of these new carriers will be superior to each other in a direct comparison and make its way into the clinic remains unknown. Nonetheless, the variety of new approaches raises hope for a more efficient therapy of inflammatory diseases.

A particularly challenging task is the delivery of GCs into the CNS while avoiding systemic exposure. To this end, a sprayable powder containing Dex-loaded microspheres blended with inert soluble carriers was recently developed and characterized in a 3D nasal cavity model [244]. In dermatology, a foam containing the steroidal compounds betamethasone and calcipotriol was tested for the treatment of eczema and found to increases penetration and bioavailability of these drugs [245]. In ophthalmology, lipid nanocapsules loaded with triamcinolone showed high stability and a significant attenuation of the inflammatory response in a rabbit model of uveitis [246]. To further improve specificity, the lipid nanocapsules were coupled to Bevacizumab, a monoclonal antibody targeting VEGF [247]. The modified nanocapsules prevented endothelial cell migration and capillary formation and improved inflammation and neovascularization. In another recent study, Dex was coupled to glucagon-like peptide-1 (GLP-1) to suppress inflammation in the hypothalamus of obese mice. With this approach, it became possible to reduce the weight gain occurring under a high-fat diet and to increase energy expenditure without causing systemic GC side effects [248].

Numerous approaches aimed to optimize GC targeting have been tested in preclinical models but hardly any of them has entered clinical trials until now. In one of the few examples, long-circulating liposomes containing prednisolone were investigated for the treatment of atherosclerosis but failed to reduce arterial wall permeability and inflammation [249]. Two clinical trials were conducted to investigate the application of GC-containing liposomes in RA therapy (NCT00241982, NCT02534896) but no results have been published up to now. Glutathion-coated MP-containing liposomes intended for the treatment of MS patients were tested for their safety, pharmacokinetics, and pharmacodynamics in a recent phase I trial (NCT02048358). Despite favorable results, however, no information is available regarding the continuation of this study [250]. Therefore, an urgent need exists to close the gap between the plethora of promising preclinical studies in animal models and translational approaches testing the new nanoformulations in clinical trials.

## 5. Conclusions

GCs have been in clinical use for the treatment of inflammatory disorders and other diseases with a presumed inflammatory contribution for more than 70 years. They show excellent therapeutic efficacy and thus remain indispensable in everyday clinical practice despite the availability of highly sophisticated biologicals such as monoclonal antibodies. Nonetheless, GC-resistance and severe side effects sometimes complicate their use, especially at high dosage and for prolonged time periods. Thus, efforts are being made to improve the therapeutic index of GCs. Analysis of different mouse models of inflammatory diseases using cell type-specific and function-selective knock-out mice carrying deletions of the GR and MR, GC-target genes, or GC-converting enzymes provided important and sometimes surprising insights into the mechanisms of GCs that are destined to stimulate various new treatment approaches. These include the development of SEGRAMs that are supposed to retain the beneficial effects of GCs while sparing at least some adverse effects, and the encapsulation of GCs in nanofomulations that allow targeting them to individual cell types. Although a plethora of preclinical studies support the great potential of these approaches, there is still a gap in translating the findings into the clinic. However, with new, exciting results emerging every day, there is hope that this old class of drug will not only continue to be used in patient care but can be further improved in the future.

## Figures and Tables

**Table 1:** Phenotype of selected genetic mouse models of neuroinflammatory diseases. MOG-EAE: EAE induced by immunization with MOG_35–55_ peptide in CFA. n.d.: not determined.

Gene	Type of Mutation	Model	Disease	Therapy (Free GCs)	Reference
GR	T cell-specific deletion	MOG-EAE	aggravated	abrogated	[47]
GR	myeloid cell-specific deletion	MOG-EAE	aggravated	unaltered	[47]
GR	impaired dimerization, ubiquitous	MOG-EAE	unaltered	unaltered	[33]
MR	myeloid cell-specific deletion	MOG-EAE	ameliorated	n.d.	[53]

**Table 5:** Phenotype of selected genetic mouse models of GvHD. Fully MHC-mismatched aGvHD model: T cell transfer from C57BL/6 mice into BALB/c mice. MHC class I-disparate aGvHD model: transfer of C57BL/6 T cells into B6.bm1 mice expressing an allogeneic MHC class I variant.

Gene	Type of Mutation	Model	Disease	Therapy (Free GCs)	Reference
GR	T cell-specific deletion	fully MHC-mismatched	aggravated	partially abrogated	[176]
GR	T cell-specific deletion	MHC class I- disparate	aggravated	n.d.	[176]
GR	myeloid cell-specific deletion	fully MHC-mismatched	aggravated	n.d.	[179]
GR	impaired dimerization, ubiquitous	fully MHC-mismatched	aggravated	n.d.	[179]

## Data Availability

Not applicable.
